# Patient related factors associated with antiretroviral therapy defaulting among the youth accessing HIV care services in Mzimba, Malawi

**DOI:** 10.1371/journal.pgph.0004507

**Published:** 2025-07-31

**Authors:** Paul Isaac Kasalu, Matthews Lazaro, Idesi Chilinda

**Affiliations:** 1 Mzimba District Hospital, Mzimba, Malawi; 2 Kamuzu University of Health Sciences, School of Nursing, Community Health Nursing Department, Lilongwe, Malawi; 3 Kamuzu University of Health Sciences, School of Global and Public Health, Lilongwe, Malawi; NYU Grossman School of Medicine: New York University School of Medicine, UNITED STATES OF AMERICA

## Abstract

Antiretroviral therapy (ART) defaulting is a serious problem among youth accessing Human immunodeficiency virus (HIV) care services in Malawi. It leads to development of drug resistance, treatment failure and increased client mortality. This study aimed at assessing patient factors influencing ART defaulting amongst youth living with HIV enrolled on ART in Mzimba District. A quantitative, case-control design was employed, enrolling 411 youths living with HIV (n = 137 cases and n = 274 controls) attending an HIV care clinic. The cases and controls were allotted to the ART clinics proportionally to their number of ART clients. Random sampling techniques were used to recruit both ART defaulters and non-defaulters. Data were collected using a structured questionnaire and analyzed using Statistical Package for Social Sciences (SPSS) version 20.0. Descriptive statistics provided counts, frequencies, proportions, and ranges, while inferential statistics established associations between dependent and independent variables. Patient related factors associated with ART defaulting include: Participants age (p-value: 0.046, Cramer’s V: 0.2, OR: 0.663, 95% CI: 0.439–0.902), forgetting appointment date (p-value: < 0.001, OR: 4.213, 95% CL: 2.289-7.755), experiencing ART side effects (p-value: < 0.001, OR: 0.438, 95% CL: 0.286-0.672), taking other drugs apart from ART (p-value 0.008, OR: 0.059, 95% CL: 0.007-0.479). ART defaulting was also associated with presence of a psychological disorder among the participants (SRQ statement 7_8) (p-value <0.001, OR: 22.119, 95% CL: 10.81-45.26) and having suicidal ideas (p-value <0.001, OR: 0.111, 95%CL: 0.051-0.241). Seventeen percent (17%) of the youths had psychological disorders, 10% suicidal thoughts, 20.0% anxiety, 12.4% felt worthless, 19.2% troubled mind, 24.8% headache and abdominal discomfort (23.8%). This study sought to identify patient factors associated with ART defaulting in Mzimba district. Participant’s age, forgetfulness, ART side effects, taking other drugs and having psychological disorders influence ART defaulting. We recommend that addressing these patient needs, may reduce defaulting to ART.

## Introduction/Background

Human Immunodeficiency Virus (HIV) remains a significant global public health challenge among the youth. The virus reduces the body’s defense against infections. The Joint United Nations Programme on HIV and AIDS (UNAIDS) announced ambitious targets indicating that 95% of all people living with HIV (PLHIV) should know their HIV status, whilst 95% of those diagnosed with HIV, should be receiving effective antiretroviral therapy (ART), and 95% of those receiving ART should achieve viral suppression by the year 2030 [1]. Despite these targets, defaulting treatment among the youth enrolled on ART program is the biggest challenge threatening achievement of these targets [2]. Evidence suggests that various patient factors can contribute to ART defaulting and they include: perceived good health, side effects, poverty, stigma and mental disorder [3–5]. These factors may vary in individuals and place.

The Malawi HIV Guidelines define an ART defaulter as an individual enrolled on treatment who fails to report for new drug supplies at 2 months when he or she is expected to have run out of ARVs [6]. Individuals that default ART, tend to develop resistance and treatment becomes ineffective if taken later. Further, the resistant strain can be transmitted from individuals infected with HIV to others [7]. It is therefore important to prevent ART defaulting.

ART defaulting is common among adolescents and youth. In 2023, about 38% of adolescents and youth had defaulted ART world wide [8]. In Sub- Saharan Africa, 24% of youth enrolled on ART program, defaulted ART [9]. ART defaulting was strongly associated with being an orphan [9]. In Kenya, more than half of the youth accessing outpatient care and treatment were documented as defaulters out of a total of 924 youth living with HIV aged 15–21 years [10]. Approximately 139 youth (26%) defaulted immediately after enrolment in HIV care in Kenya [10]. Factors associated with defaulting were non-disclosure of HIV status (AHR 1.43, 95% CI 1.10–1.89) and pregnancy during the study period (crude HR 0.68, 95% CI 0.53–0.89) [10]. Coping with physiological changes of pregnancy and ART side effects might be difficult. Another study conducted in South Africa indicated that ART defaulting was twice likely among the youth (16–24 years old) compared with older patients [11] with male defaulting treatment more than their female counterparts.

A defaulting rate of 15.5% on ART treatment was reported from January 2008 to January 2015 in Malawi [12]. A study in Zomba, Malawi that aimed at determining follow-up visit patterns in ART programs revealed that, 75.7% (n = 5,914) of all patients initiated on ART had visits which were ≥7 days late, 23.4% (n = 1,830) had visits which were ≥60 days late and 12.3% (n = 967) of patients had visits which were ≥90 days late [13]. Side effects contributed to 6.5% of defaulting among these clients. In Chiradzulo, Malawi, defaulting ART among all the clients was due to stigma (43%), care dissatisfaction (34%) and feelings that their health had improved (28%). In this study, ART discontinuation was due to: poor understanding of the disease or treatment (56%) and drug side effects (42%) [14]. However, there is paucity of literature on factors influencing ART defaulting among the youth in Mzimba district with most studies focusing on the general adult population. The District Hospital facility data indicated that 30% of the youth defaulted ART treatment in 2016 [15]. Further, data review for Mzimba District ART clinic from January- March 2019 showed that 49 youths aged between 15 and 24 years defaulted ART out of 313 clients [16]. Factors contributing to treatment defaulting have not yet been explored. The current study focused on patient-related factors to gain insights into the complexities surrounding ART defaulting among the youth and identify evidence based strategies of mitigating defaulting rates.

## Materials and methods

### Research design

We used a case control design to compare the occurrence of ART defaulting in cases and controls and to determine if there was any association with patient factors. All youths that defaulted ART were cases while controls were all youths that remained on treatment. The researcher intended to determine if patient related factors were associated with ART defaulting among this group. This was done by comparing occurrence of these factors and client’s failure to return to the clinic on the next appointment date as an outcome.

### Study setting, time and population

Participants were recruited from four ART health facilities in Mzimba district namely: the District Hospital, Embangweni Mission Hospital, Jenda and Euthini Health centers. All are public health institutions that offer services at no cost. The two hospital are secondary level health facilities whilst the health centres are primary level facilities. The researcher met the eligible participants at the outpatient department (OPD) of the ART clinics or in their homes with support from the ART provider, parents or guardians. Clients defaulting ART were followed up in their homes after obtaining their consent. A phone call was made to clients or parents and guardians using contact numbers picked from the ART cards. This enabled the researcher and data collectors to trace client’s location.

The study was conducted from 21^st^ November 2020 to January 2021. The time period coincided with Covid 19 pandemic. The pandemic led to clients shunning from services in Mzimba health facilities for fear of being tested for Covid 19. The population targeted in this study comprised of all youths living with HIV enrolled on ART treatment in Mzimba District.

#### Study setting and rationale for selection.

Mzimba District was selected as the study site due to its relevance to the firsthand experience of one of the investigators working in the district. As a healthcare professional in Mzimba, the researcher observed the challenges associated with antiretroviral therapy (ART) defaulting and was keen on identifying sustainable solutions to improve patient retention. Additionally, Mzimba is one of the largest districts in Malawi, characterized by a mix of urban and rural populations, diverse healthcare facilities, and varying accessibility to ART services. These factors provided a rich context for understanding the determinants of ART defaulting within different healthcare settings.

### Inclusion and exclusion criteria

All youths living with HIV aged 15–35 years who were enrolled in the ART program from July 2018 to June 2020 were eligible for inclusion in the study. This study included both defaulters and non-defaulters who provided informed consent. In this study, a defaulter is defined according to the Malawi HIV Guidelines, as an individual living with HIV who fails to report for new drug supplies at 2 months when he or she is expected to have run out of ARVs [6]. For those below the age of 18 years, consent was sought from their parents or guardians, and then an assent was signed to participate in the study. Excluded in this study were all youths living with HIV, diagnosed and recruited in ART programs before July, 2018 or after June, 2021. Also excluded were all youths living with HIV, on ART who were transferred out of the health facilities.

### Sample size, sampling and sampling technique

This study recruited 411 participants in a 1:2 ratio of cases to controls. Sample size for this study was calculated using a formula adapted from Charan and Biswas [17]. The required number of cases was determined to detect an odds ratio of 2.0, with a Type I error rate (α) of 0.05 and a power (1-β) of 80%. Distance to the clinic was considered the primary independent variable, with an assumed exposure prevalence of 20% among controls. Based on these parameters and a 1:2 case-control ratio, the study aimed to recruit a minimum of 137 cases and 274 controls. The sample size was allotted to the ART clinics proportionally to their number of ART clients (See Table 1).

**Table 1:** Number of study cases and controls required per health facility (n = 411).

	Total number of ART clients	Defaulters	Proportion of defaulters by health facility	Sample cases	Cases to nearest whole number	Sample controls: (cases x 2)
**Mzimba Hospital**	11066	2715 **(25%)**	70.5561331	96.6619023	97	194
**Jenda**	2326	614 **(26%)**	15.956341	21.8601871	22	44
**Embangweni**	2986	339 (11.4%)	8.80977131	12.0693867	12	24
**Euthini**	2308	180 **(8%)**	4.67775468	6.40852391	6	12
**Totals**	18686	3848 **(21%)**		137	137	274

The researcher extracted client details from the ART register, ART registration cards, and ART computer systems to locate and verify participants’ eligibility for the study. Extracted information included physical addresses, phone numbers, last clinic and pharmacy appointment dates, and upcoming scheduled clinic appointments. Data on deaths, dates when the client transferred-into and out of the clinic were also collected. Cases and controls were matched based on the health facility from which the youth accessed ARVs and their duration on ART. A two-stage cluster sampling approach was used to select the participants. The first phase that determined health facilities for the study used simple random sampling. Names of the health facilities were written on a piece of paper, folded and placed in a closed box. A volunteer was requested to draw one paper at a time, after shaking the box. Each drawn name was read aloud and recorded on an A4 sheet of paper for inclusion in the study. The process was repeated until four health facilities were selected. In the second phase, stratified random sampling was used to recruit participants (cases and controls) from the facilities in a 1:2 ratio. At each facility, two cartons were prepared (one containing identity numbers of defaulters and the other containing identity numbers of non-defaulters). To select cases, a volunteer randomly drew identity numbers from the defaulters’ carton, one at a time after thoroughly shaking it, until the required number of cases for the respective facility was achieved. The same procedure was applied to the non-defaulters’ carton to recruit controls, ensuring the target sample size for each health facility was met.

### Data collection methods, tools and data management

Data were collected from eligible participants using a structured questionnaire with close-ended questions. This instrument was designed to capture patient-related factors, categorized into socio-demographic details, physical characteristics, and psychological conditions. Socio-demographic data, including participants’ age, sex, occupation, marital status, and educational level, were recorded (Table 1). Additional information was collected on personal factors such as physiological conditions, concurrent illnesses, use of medications other than ART, and experiences of drug side effects, among others. The questionnaire also captured factors contributing to clients’ failure to attend clinic appointments as scheduled. Data collection was carried out by a team comprising of the lead author and four qualified nurses. To ensure consistency in data collection and accurate assessment of defaulting and related factors, the nurses participated in a three-day training session in quantitative data collection. All collected data were securely stored in a lockable filing cabinet located in the Maternal and Child Health (MCH) Office, with access restricted to the researcher. Electronic data were encrypted and protected with passwords known only to the researcher.

### Ethical and cultural considerations

Ethical approval to conduct the study was obtained from the College of Medicine Research and Ethics Committee (COMREC P.08/20/3102). Additional authorization was sought from the Director of Health and Social Services (DHSS) for Mzimba South and the Medical Director of Embangweni Mission Hospital. Participants were informed that their participation was entirely voluntary, and they were free to decline participation or withdraw from the study at any time without providing reasons. The study’s consent form was read to each participant, and they were requested to sign if they agreed to participate willingly. For participants under the age of 18, informed consent was obtained from their parents or legal guardians, and assent was sought from the participants. Data collection was conducted in private rooms within the health facilities to ensure both visual and audio privacy and safeguarding confidentiality. To maintain anonymity, participants were identified using codes. They were assured that their information remained strictly confidential throughout the study.

### Data analysis

In this study, data were entered into Statistical Package for Social Sciences (SPSS) software, version 20.0, for cleaning and analysis. The dependent variable for the study was ART defaulting, categorized into two levels: defaulting and not defaulting. Both descriptive and inferential statistical methods were employed. Descriptive analysis involved organizing and presenting data in frequency distribution tables. For inferential analysis, Chi-square tests were conducted by cross-tabulating the dependent variable with independent variables. Factors identified as statistically significant were candidates for using binary logistic regression to evaluate their influence on ART defaulting. A significance level of 5% was applied to all statistical tests in the study.

## Results

A total of 411 participants were interviewed from 4 health facilities namely: Mzimba district hospital, Embangweni mission hospital, Jenda Health Centre and Euthini health centre. The mean age was 24 years (SD = 1.48), with age ranging from 15 and 35 years. Median age was 22 years. The interquartile range was 11. Approximately 29% (n = 119) of the participants were males whilst 71% (n = 292) were females. Nearly half of the youths 45% (n = 185) were married, 38.2% (n = 157) were single while 13.1% (n = 54) had either divorced or separated. See Table 2.

**Table 2:** Socio-demographic characteristics of the study participants.

Variable	Frequency (%)	Cases (n = 137)	Controls (n = 274)
**Age**			
15-17 years	81 (19.7)	25(18.2)	56(20.4%)
18- 20 years	68 (16.7)	33(24.1)	35(12.8%)
21-24 years	57 (13.9)	20(14.6)	37(13.5%)
25-30 years	100 (24.3)	31(22.6)	69(25.2%)
31-35 years	105 (25.5)	28(20.4)	77(28.1%)
**Sex**			
Male	119 (29%)	45(32.8%)	74(27.0%)
Female	292 (71%)	92(67.2%)	200 (73.0%)
**Marital status**			
Single	157 (38.2%)	54(39.4%)	103(37.6%)
Married	185 (45%)	55(40.1%)	130(47.4%)
Widowed	15 (3.6%)	8(5.8%)	7(2.6%)
Separated/divorced	54 (13.1%)	20(14.6%)	34(12.4%)
**Religion**			
Christian	369 (90%)	126(92.0%)	243(88.7%)
Moslem	27 (6.6%)	9(6.6%)	18(6.6%)
Traditionalist	14 (3.4%)	2(1.1%)	12(4.4%)
**occupation**	**Frequency/Percentage**	**Cases (n = 411)**	**Controls (n = 411)**
House wife	67 (16.3%)	19(13.9%)	48(17.5%)
Farmer	68 (16.5%)	18(13.1%)	50(18.2%)
Skilled work	39 (9.5%)	14(10.2%)	25(9.1%)
Small scale business	62 (15.1%)	22(16.1%)	40(14.6%)
Student	101 (24.6%)	34(24.8%)	67(24.5%)
Unemployed	50 (12.2%)	17(12.4%)	33(12.0%)
Employed	6 (1.5%)	0%)	6(2.2%)
Other	17 (4.1%)	12(8.8%)	5(1.8%)

### ART defaulting and factors contributing to missed hospital appointments

In this study, 410 participants responded to an item that assessed if they had ever missed a scheduled clinic appointment date for ART review. Almost half of them 51.3% (n = 211) indicated that they missed a hospital appointment by either a day or more. Out of these 33.17% (n = 136) were cases and 18.29% (n = 75) were controls. Most of those who missed their appointment dates 16. 5% (n = 68) cited being too busy for a hospital visit. Other reasons mentioned were: forgetting appointment dates 12.7% (n = 52); having drugs in stock by the next appointment date 5.6% (n = 23); lack of transport 2.2% (n = 9); having travelled to South Africa to work 4.1% (n = 17) and being tired of the ART drug 1.0% n = 4) (Table 3)

**Table 3:** Reasons for missing hospital appointment date.

	Frequency (%)	Cases (n = 211)	Controls (n = 211)
Too busy to go to clinic	68 (16.5)	35 (16.6)	33 (15.6)
Forgotten appointment date	52 (12.7)	33 (15.6)	19 (9.0)
Client still had medicines on the appointment date	23 (5.6)	13 (6.2)	10 (4.7)
Was in Republic of South Africa	17 (4.1)	16 (7.6)	1 (0.5)
Lack of transport	9 (2.2)	5 (2.4)	(4 (1.9)
Spouse related challenges	6 (1.5)	6 (2.8)	0 (0)
Feeling better and do not feel like continuing with therapy	5 (1.2)	4 (1.9)	1 (0.5)
Sicknesses	5 (1.2)	2 (1.0)	3 (1.4)
Feels shy/ laughed at by friends	5 (1.2)	3 (1.4)	2 (1.0)
Tired of being on medicine	4 (1.0)	4 (1.9)	0 (0)
Confidentiality challenges	4 (1.0)	4 (1.9)	0 (0)
Taken it for too long	3 (0.7)	3 (1.4)	0 (0)
Medicine stolen	2 (0.5)	2 (1.0)	0 (0)
Travelled	2 (0.5)	2 (1.0)	0 (0)
Client not told reasons for taking ART	1 (0.2)	1 (0.5)	0 (0)
Guardian unable to read review dates	1 (0.2)	1 (0.5)	0 (0)
In police custody	1 (0.2)	0 (0)	1 (0.5)
Attending funeral	1 (0.2)	0 (0)	1 (0.5)
Missed health passport book	1 (0.2)	0 (0)	1 (0.5)
Took emergency medication	1 (0.2)	0 (0)	1 (0.5)

### Relationship between ART defaulting and patient demographic factors

A significant relationship was seen between ART defaulting and participant’s age (p-value: 0.046, Cramer’s V: 0.2, OR: 0.663, 95% CI: 0.439–0.902) including occupation (p-value: 0.048, Cramer’s V: 0.1, OR: 0.658, 95% CL: 0.434-0.997). Older ages and having an occupation were protective factors to defaulting treatment. No relationship was found between ART defaulting and marital status (p-value 0.2, OR: 0.743, 95% CL: 490-1.126) and participant’s gender (p-value: 0.219, OR: 0.756, 95% CL: 0.485-1.181).

### Relationship between ART defaulting and physical/physiological factors

An association was found between ART defaulting and forgetting appointment date (p-value: < 0.001, OR: 4.213, 95% CL: 2.289-7.755), spouse related reasons, (p-value: 0.001, OR: 6.393, 95% CL: 2.021-20.229) and client migrating to South Africa (p-value: 0.001). In this regard, the likelihood of defaulting ART were higher when the client forgot appointment date, migrated to South Africa or had spouse related reasons compared to when they did not have these. It was also observed that experiencing side effects of ART had influence on ART defaulting (p -value of 0.002, OR: 1.442, 95% CL: 0.264-2.739). Patients that had experienced side effects of ART were 1.442 times more likely to default as compared to those who did not experience the side effects. Experiencing ART side effects was a risk factor to default treatment. A significant relationship was noted with side effects such as dizziness, abdominal pains and nausea and vomiting (p-value: 0.001, OR: 2.283, 95% CL: 1.365-3.819). These side effects were a risk to defaulting treatment. In addition, a significant relationship with ART defaulting was seen where the youth were taking other drugs apart from ARVs (p-value 0.008, OR: 0.059, 95% CL: 0.007-0.479). The study also assessed specific drugs whose use alongside ARVs could result in defaulting of treatment. Antibiotic drugs (such as cotrimoxazole and contraceptives) were associated with defaulting (p-value: 0.009, OR: 0.019, 95% CL: 0.011-0.836). A more significant relationship was seen in assessing whether the client failed to take ART due to side effects experienced, (p-value: < 0.001, Cramer’s V: 0.288, OR: 0.438, 95% CL: 0.286-0.672) and whether the client failed to return to ART clinic due to the experienced side effects (p-value 0.016, Cramer’s V: 0.389, OR:1.661, 95% CL: 1.0982.512). In this case, the odds of failing to take ART due to side effects decreased by 43.8% compared to when the side effects were not experienced. On another note, the odds of defaulting ART were 1.661 times higher (66%) for clients that experienced side effects compared to those that did not experience the side effects. These results suggest that experiencing ART side effects may influence defaulting as the OR is greater than 1 on 95% level of confidence.

The other section of the study was concerned with pregnancy and breast feeding. The Chi-square test did not show any significant relationship between ART defaulting and the variable that assessed whether the client was pregnant or breastfeeding (p-value 0.947, OR: 1.019, 95% CL: 0.593-1.749). Neither was any relationship found between ART defaulting and how the pregnant or lactating young mother enrolled into the ART program (p-value: 0.428, OR: 0.012, 95% CL: 0.307-1.651).

Further analysis of the duration on ART noted a significant association with the length of time that participants had been taking ARVs (p-value 0.012, Cramer’s V: 0.164, (OR: 6.500, 95% CI: 1.407–30.037). Nevertheless, this is a weak association, suggesting that other factors may also contribute to defaulting. The study also indicated that longer duration on treatment was a risk factor to defaulting. The odds of defaulting were 6.5 times higher among participants with longer ART duration (OR: 6.500, 95% CI: 1.407–30.037). Table 4 provides a summary of results on relationship of ART defaulting and physiological factors.

**Table 4:** Relationship between ART and physiological factors.

Independent Variable	Pearson chi-square	Cramer’s V	Odds ratio	95% CL
Have you experienced any side effects of ART medicine?	0.002*	0.156	1.442*	0.264-2.739
Name of side effects experienced	0.001*	0.158	2.283*	1.365-3.819
Did you fail to take ART due to its side effects?	<0.001*	0.189	0.438*	0.286-0.672
Did you fail to return to clinic on appointed day due to side affects you experienced?	0.016*	0.389	1.661*	1.098-2.512
Are you taking other drugs apart from ARVs?	0.008*	0.176	0.059*	0.007-0.479
What other drugs are you taking apart from ARVs	0.009*	0.129	0.019*	0.011-0.836
Are you pregnant or breastfeeding?	0.947	0.004	1.019	0.593-1.749
If pregnant/lactating, how long have you been taking ARVs?	0.008*	0.278	6.500*	1.407-30.037
How did you enroll into ART program?	0.428	0.080	0.712	0.307-1.651

### The relationship between ART defaulting and psychological factors

Results from this study indicate that there was a significant relationship between ART defaulting and the presence of a psychological disorder among the participants (SRQ statement 7_8) (p-value <0.001, Cramer’s V: 0.519, OR: 22.119, 95% CL: 10.81-45.26). The odds of defaulting ART were 22.119 times higher for the youth with psychological disorders compared to those that did not have the disorder. A significant relationship was also noted between ART defaulting and all frequently occurring psychological disorder symptoms experienced by the youths. For instance, questions such as: (1). do you often have headache? had a p-value of 0.008, Cramer’s V: 0.131, OR: 0.537, 95%CL: 0.339-0.851; (2). is your appetite poor? had a p-value of <0.001, Cramer’s V: 0.322, OR: 0.163, 95% CL:0.090-0.294; and (3) do you sleep badly? had a p-value of <0.001, Cramer’s V:0.366, OR: 0.109, 95% CL:0.056-0.211. A significant relationship was also found on variables that assessed the presence of suicidal ideas in the participants (p-value of <0.001, OR: 0.111, 95%CL: 0.051-0.241). Table 4 shows the relationship between ART defaulting and variables that assessed presence of psychological disorders.

## Discussion

A case control study was conducted to determine factors associated with ART defaulting in Mzimba District. We noted that ART defaulting is associated with a number of patient related factors.

There was a significant relationship between ART defaulting and participant’s age. The odds of defaulting ART was higher among those with lower age compared to the older youths. These results are in agreement with another study that assessed the prevalence and associated factors of treatment failure among patients living with HIV and AIDS in Northwest Ethiopia [18]. In contrast, studies conducted in Zambia and South Africa indicated that the majority of ART defaulters were older clients [3,19]. The variations could be due to differences in geographical barriers [20] financial constraints and inadequate quality service packages [21] which may impact clinic visit adherence. The researcher postulates that being of young age, is a significant risk factor for treatment defaulting due to the developmental and psychosocial challenges that uniquely affect the youth. During this stage, individuals often experience identity formation, peer pressure, and emotional turbulence, which can negatively impact their adherence to treatment regimens [22,23]. These psychosocial factors, coupled with the desire for independence and potential stigma surrounding treatment, may contribute to higher rates of ART defaulting among youth [24,25]. These developmental dynamics emphasize the need for targeted interventions to support youth in maintaining consistent treatment engagement.

Youths who forgot their appointment date had a high probability of defaulting treatment than those who remembered (OR: 4.213, 95% CL: 2.289-7.755). This finding is consistent with previous studies that assessed the associated factors of treatment failure among patients living with HIV and AIDS in America [18], and Malawi [3]. This finding is contrary to a study in Nigeria which suggested that forgetfulness may not contribute to defaulting to treatment [26]. The inconsistency may be due to variations in literacy levels among the clients who defaulted. Several studies indicate that low literacy level might contribute to missing hospital appointments [27–29]. This could be due to difficulties in understanding appointment dates, inability to read or comprehend appointment reminders such as dates or tests [29]. Clients might report these as having forgotten the appointment dates. Forgetfulness may create frustration or concern for the individual on treatment. As suggested by other researchers, forgetfulness may be perpetrated by an unsupportive social system, school, work, substance use, social habits and ineffective appointment reminders [22,23]. Others have suggested that forgetfulness among clients living with HIV may be due to inflammatory processes in the brain [30,31]. A guardian or relative has been recommended to remind the youth about the return date to the health facility [32]. The current study recommends the use of multichannel client reminders such as phone calls, short message service (SMS) and community health worker visits to manage forgetfulness. In addition, the study recommends that ART providers should explore youth’s work pattern, support system and social habits to identify issues that would lead to defaulting. This could be part of an ongoing ART care to improve adherence to ART.

The youth who experienced ART side effects were found to have a significantly higher likelihood of defaulting treatment. This finding supports a study conducted in Ethiopia, which examined time of ‘lost to follow-up’ and its predictors amongst adults [33]. However, side effects of ART were not a significant reason for defaulting in a study in German [34]. The results may differ due to variations in the health systems of each country that may affect the way side effects are managed. Clients having side effects in German might have access to a wide range of drug substitutions for switching and better alternatives for managing side effects [34]. Regardless of the population studied, ART side effects present significant challenges to treatment adherence. The capacity to tolerate side effects among the youth was minimal. In Sub-Saharan Africa, side effects such as feeling dizzy were noted to be severe enough to prevent youth from leaving home or caring for themselves [35], and they failed to return for a clinic appointment [35]. Also, side effects among the youth exacerbated their emotional and psychological disorders, increasing their likelihood to default treatment [5].

In our study the use of additional medications alongside ART heightened the risk of defaulting. This finding maintains the findings by another study that assessed factors influencing adherence to ART among adults accessing care from private health facilities in Malawi [36]. The introduction of supplementary drugs such as Cotrimoxazole alongside ART may compound the burden of side effects [37], which can exacerbate difficulties in maintaining treatment. We suggests the need for healthcare workers to minimize multiple drug prescriptions to the youth on ART and to provide comprehensive support to those experiencing side effects in order to reduce the risk of defaulting.

Our study has noted that the odds of defaulting ART were higher for the youth who had a psychological disorder compared to those that did not have it. These results are congruent with another study conducted in Malawi that aimed at identifying the association between mental health status and ART outcomes [38]. Findings indicate that the presence of a mental health disorder increased the risk of defaulting ART [38]. The differences with this current study could be the age group and the methodology where the former study compared HIV outcomes and viral suppression in the populations with or without a mental health problem. The presence of psychological disorders has been noted to influence the quality of decision making [39]. Ineffective cognitive function and presence of psychiatric symptoms such as suicide, hallucinations and delusions may complicate a client’s perception towards ART [40,41]. For instance, other clients become suspicious, whilst others perceive that the ART makes them weak or they might get poisoned with it [42]. Such youth may refuse to return to the ART clinic and consequently default treatment [43,44]. Furthermore, stigma and discrimination hinders them from seeking healthcare [45]. These findings suggest the need to include mental health programs into routine HIV care for the youth to improve their psychological well-being. These could include routine mental health assessment, psychological counselling sessions and follow up.

A statistically significant association between ART defaulting and having suicidal ideas was also noted among the youth. This finding is similar to findings from a study conducted in China that assessed suicidal ideations of people living with HIV and its relationship to depression, anxiety and social support [46]. The diagnosis of HIV itself may trigger profound emotional distress, including fear, stigma, and anxiety about health outcomes [47]. All of which, may contribute to feelings of hopelessness and suicidal thoughts. Certain ARVs, particularly those in the Efavirenz class, have been associated with neuropsychiatric side effects, including mood disturbances, depression, and increased suicide ideation [48]. These effects are as a result of the interaction between the ARVs and the central nervous system, altering neurotransmitter balance and exacerbating pre-existing mental health conditions [49]. The compounded stress of managing a chronic illness such as HIV, coupled with the potential neuropsychiatric side effects of ARVs, creates a complex landscape where clients may feel overwhelmed, particularly in the absence of strong psychosocial support [50,51]. Effective management of this risk requires an integrated approach that includes mental health screening, close monitoring of ARV side effects, and timely interventions through counseling and psychiatric care to mitigate the risk of suicide ideation among clients living with HIV. Addressing these challenges holistically can improve both mental health outcomes and non-defaulting to life-saving ARV therapy.

## Strengths and limitations

The sample size was large enough to increase representation of study participants to the population under study. The application of a two-stage cluster random sampling method ensured a broad and diverse participant selection from all health facilities within Mzimba district. However, since data were gathered solely from Mzimba South, the ability to generalize these findings to other settings may be constrained. Therefore, the study results should be interpreted with caution. Despite this, the study’s internal and external validity remained intact and were not significantly compromised.

## Conclusion

This case control study identified several patient-related factors that are associated with ART defaulting among youth in Mzimba district. Forgetfulness, experiencing ART side effects, use of additional medications, and psychological disorders significantly contributed to defaulting. Forgetting clinic appointments due to unsupportive social systems or cognitive impairments was linked to missed drug refills, leading to defaulting. Side effects, especially when compounded by additional medications, exacerbated ART defaulting. Psychological disorders, including suicidal ideation, further hindered retention in ART program, emphasizing the need for mental health integration into HIV care among the youth. Addressing these factors through comprehensive support systems and mental health screening are suggested to reduce ART defaulting among the youth.
